# COVID-19 and democracy: a scoping review

**DOI:** 10.1186/s12889-023-16172-y

**Published:** 2023-08-30

**Authors:** Ville-Pekka Sorsa, Katja Kivikoski

**Affiliations:** Faculty of Social Sciences, University of Helsinki, P.O. Box 18, FI-00014 Helsinki, Finland

**Keywords:** COVID-19, Democracy, Resilience, Scoping review

## Abstract

**Background:**

The resilience of democracy is tested under exogenous shocks such as crises. The COVID-19 pandemic has recently tested the resilience of democratic institutions and practices around the world.

**Aim:**

The purpose of this article is to scope the early research literature that discusses democracy and the COVID-19 pandemic. We review scientific journal articles published during the first two years of the pandemic. We ask three research questions in scoping this body of literature: (1) what are the key topic areas of all published research that associates itself with both democracy and COVID-19, (2) what kinds of conceptual and theoretical contributions has research literature that more specifically discusses democracy under the pandemic produced, and (3) what are the impacts of democracy to the pandemic and vice versa according to empirical research?

**Methods:**

The scoping review methodology draws on systematic literature search strategies, computational methods, and manual coding. The systematic Web of Science search produced 586 articles for which we conducted a Correlated Topic Model. After technical and manual screening, we identified 94 journal articles that were manually coded.

**Results:**

The early research on democracy and the COVID-19 pandemic offers a versatile body of scholarship. The topic modeling shows that the scholarship discusses issues of crises, governance, rights, society, epidemiology, politics, electorate, technology, and media. The body of papers with conceptual and theoretical contributions has offered new insights on the difficulties, possibilities, and means to maintain democracy under a pandemic. Empirical research on democracy’s impact on the COVID-19 pandemic and vice versa varies in terms of methodology, geographical scope, and scientific contributions according to the direction of influence studied. Democracy appears to have a significant impact on some aspects of policy responses and epidemiological characteristics of the pandemic. In most parts of the world, the scope, franchise, and authenticity of democracy narrowed down due to the pandemic, albeit in most cases only temporarily.

**Conclusions:**

A significant number of papers show that the pandemic has accentuated democratic backsliding but is unlikely to have undermined established democracies that have proved resilient in face of the pandemic. But empirical research has also made visible some weak signals of antidemocratic tendencies that may become more accentuated in the longer run.

## Background

Democracy is inextricably linked to crisis [1]. Democracies are often perceived to be in crisis due to the absence of some features which we consider as definitional of democracy [2]. While different theories of democracy may focus on the absence of different features, most would agree that exogenous shocks, especially large-scale crises, such as financial crises or pandemics, are the key factors that challenge and test the durability of democratic institutions and practices. Democracy rarely flourishes under large-scale crises and crises tend to have negative impacts on democracy; but democracy may as well recover, revive, and sometimes even strengthen after crises [2].

The resilience of democracy in face of external shocks has recently gained much research attention [3]. Democratic resilience can be defined as the capacity of democratic institutions and practices to absorb and recover, adapt, innovate, or transform in response to shock or crisis. Democracy is a contested concept, both an ideal and a system of government, and inclusive of procedural and substantive elements, and ultimately about collective decision-making, which means that democratic resilience inherently lacks the conceptual specificity of resilience as it is found in some other disciplines [3]. As a political form, democracy is the perpetual absence of something more, an always pending agenda that calls for the redress of social ills and further advances in the manifold matters [4]. This means that there are neither external safeguards for democratic politics nor any kinds of internal guarantees that democracy will be maintained under crises: democracy survives crises only if citizens continue to engage with democratic politics under them. Hence, the impacts of crises to democracy depends on how much of the society is kept under democratic control, to what degree people rely on democratic institutions to solve conflicts and problems, and to what degree people participate in democratic politics in the crisis conditions [2].

No crisis has recently tested democracy as much as the COVID-19 pandemic [5, 6]. The pandemic has had enormous impacts to the society, economy, health systems, and everyday lives globally. The cause of the pandemic, the new severe acute respiratory syndrome coronavirus 2 (SARS-CoV-2), was first identified from an outbreak in Wuhan, China, in December 2019. The World Health Organization (WHO) declared a Public Health Emergency of International Concern on 30 January 2020 and a pandemic on 11 March 2020. The COVID-19 pandemic has also given rise to an exceptionally broad and rapidly growing body of research in various scientific disciplines and numerous fields of research. The research has also been followed by an exceptionally large number of published literature reviews. The high number of reviews can be in part explained by the necessity to synthesize findings for policymaking purposes [7].

Our scoping review is the first attempt to scope early research on democracy and the COVID-19 pandemic. We combine semi-systematic and integrative approaches to the scoping task. Our review combines systematic search strategies, computational methods, and manual coding. The scoping review covers all scientific articles included in the Web of Science database by the end of March 2022. The period studied covers roughly the first two years of the pandemic, which saw the rise and, towards the end of the period, the decline of wide policy responses around the world. The body of literature discussed here represents only early research on the topic, and much more research is likely to be published on the topic in the near future. Scoping the research published during this period is important, as this literature has, at least in principle, been available for policy experts, policymakers, and public officials during the acute phases of the pandemic. We are thus scoping the peer-reviewed research that has *potentially* had an impact on the policy and politics of the pandemic response and, possibly, the maintenance of democratic politics in crisis conditions.

We focus on three issues in our review. First, using computational methods, we scope the topics of published scholarship that associates itself explicitly with democracy and the COVID-19 pandemic. Second, we scope the conceptual and theoretical contributions of research literature that more specifically discusses democracy under the pandemic. Third, we scope the empirical research on the impacts of democracy to the pandemic and vice versa.

## Methods

### Scoping reviews and democracy research

The purpose of a scoping review is to map a complex research field [8]. Unlike systematic reviews that seek to synthesize evidence on clearly defined topic or phenomenon, scoping reviews seek to scope the topics of a body of literature, clarify concepts, identify knowledge gaps, or/and to investigate research conduct [9]. Scoping review is viewed as helpful for understanding complex research fields that are in nature highly heterogeneous and fast growing [10]. Scoping reviews that define a field in this way have been considered especially useful in political sciences [11]. Scoping reviews that deal with highly complex fields can deploy various review methods [9]. Typical to scoping reviews is to combine elements of semi-systematic and integrative review methods [12].

Democracy qualifies as a highly complex and heterogeneous research field. The concept of democracy is essentially contested and deployed differently by schools of thought differentiated by various normative, philosophical, and theoretical commitments [13]. Research on the COVID-19 pandemic qualifies as a fast-growing field. The evolution of the COVID-19 pandemic over time and variation and change in government responses to the pandemic suggest that the complexity of the research field may also have increased over time.

It is important to note here that two ‘academic worlds’ of democracy research exist [14]; one of theoretically grounded (positive) empirical research into real-world democratic, democratizing, or non-democratic regimes, institutions, and practices; and another of political philosophy that critically and normatively assesses different conceptions and (possible) practices of democracy. In the former world, the configurations of political organization that can be called ‘democratic’ and the research objects addressed by research depend on the theory or model of democracy onto which the research is founded. This research can be approached with systematic and semi-systematic review strategies. In the latter world, the concept of democracy is deployed to discuss a broad variety of theoretical and normative issues in various topic areas that range far beyond political systems, institutions, and practices [15]. An unclearly bounded, conceptually moving, and often incommensurable field easily escapes systematic review methods, and requires more interpretive and integrative methods [11].

Like semi-systematic scoping reviews, we ask broad research questions, use systematic search strategies, and focus only on scientific articles to recognize key themes and assess the state of knowledge in early research on the topic. But, like integrative scoping reviews, we are not focused on the details of research conduct but seek to scope and, where possible, synthesize the key approaches, insights, and arguments presented in research. Identifying the key topics of the ‘second world’ research would be laborious with solely manual coding. Therefore, we use computational methods that can deal with a large body of research to assist our work. This review strategy allows us to scope the key topics of both the ‘first world’ and the ‘second world’ of democracy research as well as to provide more nuanced scoping of the findings and contributions of research in the former ‘world’. Our review is conducted in three stages: collection and screening of the sample, computational topic modeling, and manual coding (see below).

### Research questions and limitations

Our review is guided by three research questions:

1. What are the key topics of research that deploys the concepts of democracy and the COVID-19?
2. What kinds of conceptual and theoretical contributions has been produced by research that discusses democracy under the pandemic?
3. What is the mutual relation between democracy and the COVID-19 pandemic according to empirical research: how has democracy affected the pandemic and how has the pandemic affected democracy?

The main limitation of our review is that we scope only the topics under which democracy and the pandemic is discussed, and the key insights and arguments (e.g., conceptualizations, theories, empirical findings, normative assessments) that have been presented. Due to the high proportion of commentaries, essays, and conceptual papers it would add limited value to discuss empirical research settings in detail. We discuss issues of research conduct only by identifying the broad research strategies and key indicators used and geographical areas addressed in empirical research. We leave the issue of more detailed research design and methods for later systematic reviews. Another limitation is that we exclude from our review the monographs and edited volumes that have been published on the topic during this period (e.g., [16–18]). These publications include complex arguments that rely on highly varied conceptualizations of democracy and scopes of analysis. Their summarization requires thematically more focused research questions and integrative review methods.

### Sample and technical screening

We conducted a search in the Web of Science database with a simple search string *covid-19 AND democracy* addressed to all search fields to identify relevant terms for more focused searches. We used the term ’covid-19’ to focus only on the specific pandemic and exclude previous pandemics from the query. We addressed all search fields to allow for the possibility that another term would be used in the title or abstract. The search included all items indexed in the databases by 31 March 2022. The search produced a body of 617 items. We excluded from this body all other items than published journal articles (such as unpublished conference presentations, posters and working papers), one retracted article, and all articles without an abstract. With these measures, we were left with 586 articles, which we use as a sample for answering our first research question through topic modeling (see below).

A quick reading of the article titles suggested that most items were unlikely to address democracy or/and the pandemic in a way that provides answers to our second or third research question. The reading also suggested that some referrals to the pandemic used different terms for COVID-19 in the title and main text. We changed the search string on the COVID-19 to ‘covid’ OR ‘pandemic’ OR ‘current public-health emergency’ for our further screening. We then deployed various technical measures to narrow down the corpus. We included only articles that explicitly used search terms democra* and covid-19/pandemic/current public-health emergency in the title or/and abstract. We thus use the inclusion of a key concept in the title or abstract as a proxy for an explicit discussion of the concept or some phenomena described by it, and the inclusion of two concepts as a proxy for explicit discussion on some relation between the two concepts or some phenomena described with both concepts. We also included in the review only articles whose main text was in English. With this screening, the items narrowed down to 383 articles. This body of literature comprises of all research that can potentially answer our second and third research questions.

The works excluded by this measure include some publications that discussed closely related topics to the publications included in our review but did not associate the topic explicitly with democracy. These include legal studies on the constitutional aspects of government responses to the COVID-19 pandemic, political science scholarship addressing changes in power relations during the pandemic, and multidisciplinary studies on human rights in connection with the pandemic. Even though these publications are excluded from our review, they may provide some additional insights on topics that other scholars have explicitly associated with democracy. Addressing these studies in further reviews requires more focused systematic reviews.

### Topic modeling

We conducted a topic modeling for the 586 items to generate an initial understanding of the contexts in which democracy and the COVID-19 pandemic have been simultaneously mentioned by a wide body of research. We implemented Correlated Topic Model (CTM) [19] using Structural Topic Model (STM) package in R to study the topics in the abstracts of the 586 articles. In case of no covariates used, the STM model reduces to implementation of CTM [20]. Common pre-processing steps were taken, including removal of punctuation, stopwords, and numbers, followed by the removal of infrequent terms which only appeared in maximum of one document. We did not use stemming, as it is shown that stemmers produce no meaningful improvements for the process [21].

To select the number of topics, we ran the model first with k = 5, 10, 20, 30, 40 and 50. On the second run we narrowed the scope to k = 5–20. From the second run we chose the best number of topics to be 9. To select the best model, we estimated a set of 20 separate 9-topic CTMs with different initializations. The model selected involves a trade-off between maximized semantic coherence and exclusivity of the topic-word vectors, which is typical to model selection [20]. We explored the estimated topics using the words associated with each topic, and then validated the topics by selecting one document with the highest document-topic loading per each topic and manually checked the abstract and title of those documents. All steps in the topic modeling process were performed using R. We report our findings in the next section of the paper.

### Manual screening and coding

Next, our analysis shifted to the 383 articles. We screened the articles manually in two phases: first through abstracts and then through full texts. The purpose of the screening was to identify the articles that provide conceptual or theoretical contributions to democracy research (second research question), or/and discuss the impacts of democracy to the pandemic or vice versa (third research question). In both phases we worked with Rayyan software [22]. Due to the complexity of the topic at hand, we did not use blinded decision-making regarding inclusion/exclusion. This allowed us to reflect upon the decisions as a team. Conducting an unblinded review has not been found to increase the risk of bias in systematic reviews if review decisions are documented [23]. We documented our discussions as notes in the Rayyan software.

In the first phase, screening required very little interpretation. For example, several publications were omitted on the basis that they studied something that occurred in a ‘democratic’ or ‘non-democratic’ country during the pandemic but did not otherwise address democracy or associate their more specific research object with democracy. More interpretation was needed in two specific types of cases. First, we excluded articles in which the publication presented democracy or the pandemic as a contextual factor but did not address it directly. Excluded articles typically studied something that took place during but not due to the pandemic or/and presented their findings as a potential but not (at least yet) actual concern for democracy. We used labels ‘covid-19 not the research topic’ and ‘democracy not the research topic’ in Rayyan to mark these publications. Second, we excluded papers that addressed issues explicitly associated with democracy and the pandemic but did not discuss either of the two directly. Articles that treated both democracy and the pandemic only as covariates for something else were also excluded here. We used labels ‘indirect link to the pandemic’ and ‘indirect link to democracy’ in Rayyan to document these exclusion decisions.

124 articles remained after manual screening of the abstracts. In the second stage, our attention turned to the full texts to screen and code the remaining articles. The measure excluded 30 articles. The main reason for excluding an article at this stage was that the association with democracy or the COVID-19 pandemic in the abstract proved indirect in the full text. For example, the full text of one article [24] shows that the article explicitly discusses liberalism and *not* democracy under the pandemic, even though the term ‘liberal democracy’ is used in the abstract. Another reason for exclusion was that the use of the term ‘democracy’ (or some of its variation) in the abstract proved as a synonym or label for something else in the full text without addressing its democratic qualities or “democraticness”. For example, one paper used the label ‘democratization’ explicitly as a synonym for widening user/patient involvement without other references to democracy [25].

Our final sample for answering the second and third research question includes in total 94 articles, which are all listed in the references. We used the topics produced by topic modeling to provide preliminary labels to each article in Rayyan. We added labels to divide the articles to three broad categories: (1) all articles with conceptual, normative, and theoretical contributions (second research question), (2) empirical articles discussing democracy as a determinant of some aspects of the COVID-19 pandemic (first part of the third research question), and (3) empirical articles discussing the pandemic’s impacts on democracy (second part of the third research question).

For each of the three broad categories we gathered key insights from the articles into a separate Excel table. We first collected the conceptual and theoretical contributions regarding the COVID-19 pandemic to one table. In case of the two other broad categories, we split tables by thematic labels. We used two initial labels under the second broad category – epidemiology and policy responses – which ultimately covered all articles in the broad category. The challenge with the third broad category was that most articles deployed different models or conceptions of democracy; hence, we needed labels that address change but can be used independent of the model or conception of democracy. We used Dryzek’s [26] notions of franchise, scope, and authenticity of democracy (see below) as our initial labels. We then collected the key aspects of democracy addressed or/and democracy indicators used, geographical focus areas, and findings and/or conclusions of each paper to the tables.

## Results

### Topic modeling

We conducted a CTM on the abstracts of the 586 articles to scope the topics of research that mentions the COVID-19 pandemic and democracy. Nine topics offered the most consistent account in terms of semantic coherence and word-topic exclusivity trade-off. The nine topics were relatively equal in proportion. The most common topic was expected to appear in 14.2 per cent and the least common in 8.5 per cent of the abstracts. We interpreted the most probable and frequent and exclusive (FREX) terms to label the topics. We used R to randomly select articles whose abstract involves the topic to validate the labels.

**Table 1:** CTM results and topic labels

Topic label	Topic proportion	Most probable terms	Frequent and exclusive terms (FREX)
Crisis	14,2%	democratic, crisis, pandemic, democracy, state, covid	union, south, democratic, european, disaster, executive, central
Governance	13,1%	public, health, pandemic, covid, governance, policy, global	governance, science, public, swedish, leadership, argue, challenges
Rights	12,9%	rights, pandemic, human, covid, international, democracy, law	courts, autonomy, rights, plague, court, human, athens
Society	12,2%	social, new, pandemic, covid, democracy, food, economic	neoliberal, energy, civic, urban, food, inequality, youth
Epidemiology	10,2%	covid, countries, data, pandemic, higher, study, democracy	rates, infected, fatality, deaths, infections, index, per
Politics	9,9%	political, covid, pandemic, change, social, crisis	hate, speech, populist, chinese, surrounding, coalition, opposition
Electorate	9,6%	pandemic, covid, trust, elections, support, political, government	voters, attitudes, survey, perceived, trust, electronic, elections
Technology	9,5%	covid, digital, health, use, data, pandemic, social	employees, technologies, remote, vaccine, surveillance, digital, hesitancy
Media	8,5%	media, covid, news, information, social, study, crisis	news, fake, conspiracy, tweets, false, journalists, rumors

The labels of the topics are listed in Table 1. Some clarification is needed. We labelled the most common topic as ‘crisis’. Common to the abstracts of this topic is to represent something as (or as being in) a crisis. While the topic includes explicit discussions on the crises of democracy caused the pandemic, it also includes many other discussions such as the legacies of previous (political, economic or health) crises and their implications to the functioning of democracy under or policy responses to COVID-19. We labelled the second most common topic as ‘governance’, as the term was among the most frequent and expected in this topic. However, as the topic includes numerous articles that deal with politics, policies and leadership related specifically to national policy responses to the pandemic, it could be also titled ‘policy response’. We used this latter term later as a label in manual coding.

Three topics are relatively homogenous. The topic ‘rights’ largely revolves around issues of human rights during the pandemic, the topic ‘epidemiology’ around statistics regarding the pandemic, and the topic ‘media’ around social media and news. Two of the topics are much more heterogeneous. The topic labeled ‘society’ discusses a variety of civic and economic issues raised by the pandemic. Common to these is the highlighting the importance of non-state actors; hence, ‘society’. The topic labeled ‘technology’ discusses highly varied issues such as population surveillance, vaccine development, and remote work in equally varied contexts. Common to these issues is the concerns caused by the rapid adoption of new technologies under the pandemic; hence, ‘technology’.

### Conceptual and theoretical contributions

The breadth of conceptual contributions varies in the scholarship reviewed. Broader conceptual contributions to democracy scholarship can be found in individual conceptual papers (see Table 2) and special issue introductions [27, 28], while critical assessments and essay articles typically address some more specific and limited aspects of democracy. Some have asked how the pandemic may have changed the conceptions of democracy [29]. As expected from early research on any topic, the body of literature involves few syntheses of research on the state of democracy under COVID-19. The notable exceptions here are Hellmeier et al. [30], who seek to synthesize the impacts of the COVID-19 pandemic to democracy through a comprehensive analysis based on the Liberal Democracy Index of the Varieties of Democracy (V-Dem) dataset, and Afsahi et al. [27], who build a synthesis of 32 democracy scholars’ early insights on the impacts of the pandemic to democracy.

**Table 2:** Articles with conceptual and theoretical contributions

Article	Article type	Topic area	Democracy focus	Conclusions
Afsahi et al. (2020)	Special issue introduction	Synthetization of 20 articles and 32 experts’ views on how the COVID-19 pandemic has impacted democracy	Democratic institutions and democratic performance	COVID-19 has had corrosive effects on already endangered democratic institutions, revealed alternative possibilities for democratic politics in the state of emergency, amplified the inequalities and injustices within democracies, demonstrated the need for institutional infrastructure for prolonged solidarity, and highlighted the predominance of the nation-state and its limitations.
Alon et al. (2020)	Conceptual article	A preliminary comparison between democracies and authoritarian regimes in their responses to COVID-19	Four key elements of democracy: elections, participation, human rights, rule of law	Democracies are not intrinsically inferior to authoritarians in crisis response. Authoritarianism is not a prerequisite in dealing with the coronavirus or other crises.
Bar-Siman-Tov (2020)	Conceptual article	Analysis of the multiple ways in which the pandemic challenges legislatures and their operation.	Assembly of people as enactment of democratic values and purposes; appropriate use of emergency powers	COVID-19 poses a unique and complex challenge for legislatures resulting from the characteristics of this pandemic and the ways they interact with the fundamental institutional features of legislatures.
Boschele (2021)	Review article	The ways in which Western democratic governments have responded to the crisis and the way normative values and ideas have influenced the pandemic policy.	Democratic accountability and expert pluralism	The COVID-19 crisis further highlighted the long-standing tensions between technocracy and democracy.
De Angelis and de Oliveira (2021)	Conceptual article	Assessment of the institutional resilience of consolidated democracies in emergency situations	Restrictions of rights, freedoms, and access to documents; legal basis of and legal acts accompanying emergency measures	Seven criteria for assessing the democraticness of the declaration of state of exception
Edgell et al. (2021)	Conceptual article	Conceptualization of democratic standards for emergency measures.	Disproportionate, non-necessary, or discriminatory derogation of human rights	PanDem index: seven types of violations and 15 indicators
Goetz and Martinsen (2021)	Special issue introduction	Dual challenge to democratic principles and democratic performance that the COVID-19 pandemic has posed to European liberal democracies	The governance of emergencies and of emergency politics; political turbulence and organisational and policy responses	Assessments of the likely longer-term effects of COVID-19 on the principles and performance will need to draw on both sectoral and systemic perspectives, with a focus on the organisation and operation of public authority and the state.
Greedy (2020)	Essay article	Human rights and transparency under COVID-19	Human rights	Requirements for policy responses: transparency and accurate information about risk, transmission, and treatment; active engagement of populations; and maintaining a climate that will encourage people at risk or ill to seek help.
Greer et al. (2020) [106]	Conceptual article	Understanding policy and politics as determinants of different responses to COVID-19 and their effects.	Binary regime (democracy or autocracy)	Research agendas to address the COVID-19 pandemic that takes politics as a serious focus.
Greitens (2020)	Conceptual article	To what extent has the COVID-19 outbreak and the augmented use of health surveillance technology altered conceptions of civil liberties, privacy, and democracy	Privacy and citizen rights	Use of surveillance in consolidated democracies has been fenced in by democratic institutions and rule of law. Weak democracies exhibit some risk of democratic erosion and autocratization, but surveillance has played a limited role here.
Grogan (2022)	Conceptual article	Preliminary analysis on how the global health crisis affected the state of democracy and the rule of law	Various democratic institutions	COVID-19 measures were often uncertain in their meaning, arbitrary in their application, and of questionable basis in the law. Oversight was often limited or lacking in many states, and the laws introduced during the pandemic risked causing permanent shifts in the balance of power towards the executive.
Haagh (2020)	Conceptual article	How the COVID-19 crisis has brought to light the importance of state democratic capacities linked with humanist governance.	Institutions that protect and promote individuals’ control of their lives	Moving democratic theory beyond the concern with redistributive and participatory features of democracy to consider foundational institutional properties of democratic deepening and freedom in society.
Hellmeier et al. (2021)	Conceptual article	The state of democracy in 2020.	Principles of liberal democracy (46 V-Dem indicators)	The direct effects of the pandemic on levels of liberal democracy were limited in 2020. The threat to freedom of expression is intensifying. Due to the pandemic and state restrictions on the freedom of assembly, mass mobilization declined to its lowest level in over a decade.
Hsieh et al. (2021) [107]	Conceptual article	How liberal democracies can control and counteract COVID-19 without resorting to authoritarian methods of containment.	Balancing of public health and individual rights	Democratic outbreak control can succeed only if there is an integrated system of interdepartmental, central-local, intersectoral and citizen-state collaboration.
James (2021)	Conceptual article	Organizational ‘elephant traps’ that polities will need to side-step during pandemics to safely protect the healthy running of elections.	Safe elections	In order to secure electoral integrity governments, legislators, and electoral management bodies need to build political consensus, consider the impact on the whole electoral cycle, include a wide range of stakeholders in meetings, invest in sufficient resources, undertake risk assessments, and avoid late major changes to electoral law.
James and Alihodzic (2020) [108]	Conceptual article	Postponement of elections	Electoral integrity	The decision of whether to postpone or hold an election should be subject to assessment against broader democratic theory rather than international law and standards.
Katner et al. (2020) [109]	Conceptual article	The failure to respond effectively to the pandemic in the US.	Representation and accountability	Steps helping to establish trustworthy democratic representation to prepare for and ideally seek to prevent future disasters.
Kavanagh and Singh (2020) [110]	Conceptual article	Comparison of pandemic response and population health.	Democratic mechanisms for improving health: incentives, information, accountability, and association	Several of the mechanisms through which democracy has been shown to be beneficial for health have not traveled well to explain the performance of governments in this pandemic.
Keen (2021) [111]	Conceptual article	How democracies may struggle to confront disasters that are increasingly impinging on the Global North.	Democratically elected policymakers’ perceptions of disaster, state-market-relations, free speech	A key problem in the UK and the US is that these countries were not democratic enough.
Kortum et al. (2020)	Conceptual article	User-centered voting systems that support the safe conduct of voting in a pandemic environment.	Safe elections	Potential solutions: vote by mail, safer polling station practices, outdoor voting, drive-through voting, ballot drop-off voting, internet voting
Kövér (2021)	Special issue introduction	Government - civil society organization (CSO) relations in the pandemic	Autonomy, participation, and solidarity as democratic qualities of civil society	Various assessments of the democraticness of government-CSO-relations in different countries
Landman and Splendore (2020)	Conceptual and theory article	Assessment of the risks posed by the pandemic on the conduct of genuine and transparent elections in the world	Organization of and participation in elections	The virus can discourage voters from casting their votes and affect overall levels of turnout. The consequences of formal postponement varies by regime type. Many different elements in the electoral cycle may be affected.
Lo and Shi (2021) [112]	Conceptual article	Questioning of the “liberal democracy versus authoritarianism” dichotomy.	Political establishments’ accountability to and representativeness of the people	With competition as both its guiding principle and functionality, the US political system appears to be handicapped in handling the epidemic crisis.
Mohee (2021) [113]	Conceptual article	Investigation of the threats posed to democracy, the rule of law and human rights as experienced in Africa since the outbreak.	Governance and conduct of elections	Perennial tactics of political repression and crackdowns on civic space continue to characterize the African electoral landscape and are further amplified by the ostensible prioritization of public health concerns.
Morrissey and Rivera-Agosto (2021) [114]	Essay article	The power of constituencies to influence policy deliberation in a democracy under pandemics.	Open and participatory dialogue	The work done in New York may serve as a model for other states in public health planning and research for the purposes of developing policy reforms.
Nikolova (2021)	Conceptual article	Identification of two dangers for democracy that emerge from the failures of the current governance paradigm	Balance of powers and social distance	COVID-19 is testing the resilience of crucial components of democratic governance such as the right of assembly, of public gathering, of protest, and civil disobedience.
Parry et al. (2021)	Conceptual article	Demonstration of how a systemic view of democracy can provide insights into the ways in which the pandemic affects democracies worldwide	Participation and deliberation	Public space has been partially relocated into private space. Empowered space has flexed its limbs into private space through executive rule and surveillance. Within empowered space, the executive has further expanded its power over the legislature.
Peng and Berry (2021)	Conceptual article	Assessment of the negative and positive outcomes of the pandemic	Freedom of movement and privacy	The current tools and technologies used for disease surveillance and some of the aggressive measures for disease control may pose threats to democracy and personal privacy
Rapeli and Saikkonen (2020) [115]	Essay article	Discussion on some possible effects of the pandemic in established and newer democracies.	Democratic insitutions and leaders and their support	We expect that the pandemic will not have grave long-term effects on established democracies, the repercussions of the pandemic can aggravate the situation in countries that are already experiencing democratic erosion, and the long-term economic effects of the pandemic may be more detrimental to non-democratic governance.
Schrager (2021) [116]	Essay article	Problematic trends that hinder the capacity for democracies to respond to present and future crises.	Democratic legitimacy of scientific expertise	COVID-19 is new, but it intersects with the vexing challenges that confront democratic governance.
Stevens and Haines (2020)	Essay article	Citizen and civic behavior brought into being by TraceTogether app	Participation and transparency	Rather than fostering citizen empowerment, engagement, or democratic participation, TraceTogether is ultimately a technology that encloses and centralizes data.
Thomson and Ip (2021) [117]	Conceptual article	The regression of governance to authoritarianism triggered by the invocation of public health emergency powers.	Human rights, appropriate use of emergency powers, democratic control over governments	There are unmistakable regressions into authoritarianism in governmental efforts to contain the virus.
Weiffen (2020) [118]	Essay article	The immediate repercussions of the crisis for democracy in Latin America.	Political rights	In countries already affected by democratic erosion, leaders might be tempted to take advantage of the crisis and prolong instruments such as the state of emergency to do away with obstacles to their rule.

Some articles conceptualize and specify the social and political conditions of a large-scale pandemic. One paper introduces an index to capture these conditions. Edgell et al. [31] have constructed The Pandemic Violations of Democratic Standards Index (PanDem) to assess the extent to which states have violated different types of human and political rights during the pandemic. The index is used widely in empirical research (see below). Another paper introduces a new conceptual approach to democracy to capture these conditions. Parry et al. [32] describe a ‘systemic view of democracy’ that conceptualizes how participation and deliberation has occurred across private, public, and empowered spaces of communication and contestation under the pandemic. While some have conceptualized different aspects of the COVID-19 pandemic from the perspective of democratic theory [33, 34], others have discussed the qualities of some aspects of democratic politics in the conditions of a large-scale pandemic [35–38]. Some have also discussed whether democracies are intrinsically inferior or superior to autocracies in dealing with pandemics [39].

The rest of the papers in this category conceptualize and critique some specific challenges or dangers that the COVID-19 poses to democracy. Nikolova [40] focuses on the legitimization of imbalances of powers and normalization of social distance as having negative effects on democracy, while Peng and Berry [41] focus on the negative impacts of the pandemic to freedom of movement and privacy. Otherwise, the focus ranges from macro-level issues such as government-civil society relations [42] to micro-level case studies such as particular surveillance technologies [43]. Some, but not all papers, provide prescriptions for dealing with the outlined challenge [44]. While most papers in this category are essays or conceptual papers with empirical examples, one review article also exists on democratic accountability [45].

### The relations between democracy and the COVID-19 pandemic

Next, our attention turns to empirical research on the relations between democracy and the pandemic. Three key differences exist in this research depending on the direction of influence studied. First, the research strategies. Research on democracy’s impact on the pandemic is almost exclusively statistical, and typically based on different democracy indices (see Table 3) and either pandemic response indices or epidemiological statistics. Few articles that address the other direction of influence uses democracy indices. The articles addressing this direction are mostly descriptive and methodologically varied, typically deploying interpretive methods to assess the quality of impacts.

Second, the geographical scope of research varies between the two directions. Most research on democracy’s impact on the pandemic is based on global comparisons with more than 100 countries included in the analysis. The findings of the few articles that focus on a narrower group of countries offer somewhat different findings from these. Very few articles focus on the sub-national level and more in-depth comparisons of individual countries. In contrast, the research addressing the pandemic’s impact on democracy largely addresses individual countries or comparisons of relatively few countries.

Third, the scientific contribution of the published scholarship varies according to the direction. Few papers addressing democracy’s impact on the pandemic aim at theory-building. Few papers address or specify the mechanisms and processes through which democracy has tangibly influenced the pandemic in the cases studied. Due to the extensive reliance of this research on the existing indices, much of the future theory-building work to which this research may contribute is thus limited to factors that can be accommodated to the most popular indices. In contrast, most papers that address the pandemic’s impact on democracy engage with in-depth cases and pursue wider theoretical contributions with them.

**Table 3:** Democracy indices used in the reviewed research

Source	Total	Index	Total
Economic Intelligence Unit	10	EIU	10
Freedom House	6	Civil Rights Index	1
		Global Freedom Score	3
		Political Rights Index	1
		Political Rights Rating	1
Polity	4	Polity (undefined)	1
		Polity IV	2
		Polity2	1
The Swiss National Center of Competence in Research	1	The Democracy Barometer	1
Varieties of Democracy (V-Dem)	24	Egalitarian Democracy Index	1
		Electoral Democracy Index	4
		Liberal Democracy Index	5
		Multiplicative Polyarchy Index	1
		Pandemic Backsliding Index	1
		Pandemic Violations of Democratic Standards Index	3
		Participatory Democracy Index	1
		Physical Violence Index	1
		Political Civil Liberties Index	1
		Private Civil Liberties Index	1
		Regimes of the World	2
		Rule of Law Index	3

### The impacts of democracy to the COVID-19 pandemic

#### Policy responses

Most studies addressing the impacts of democracy to pandemic responses draw their indicators from various democracy indices, The Oxford Covid-19 Government Response Tracker (OxCGRT), or/and the PanDem index. Most analyses discuss a wide group of countries. Some single country analyses [46] and sub-national analyses [47, 48] also exist (see Table 4).

**Table 4:** Articles discussing the impacts of democracy to policy responses

Authors (year)	Geographic location	Topic area	Indicators / explanandum	Indicators / explanans	Conclusions
Chathukulam and Tharamangalam (2021)	India (Kerala)	Examination of trajectory in achieving the success in three waves of COVID-19	Sufficient consensus to provide proactive interventions	Social mobilization and participation, state-society collaboration	States that have handled the crisis well have relatively effective models of social democracy.
Chen et al. (2021)	Global (152 countries)	Institutional and cultural determinants of the speed of government responses during the COVID-19 pandemic.	Marginal rate of stringency index change (OxCGRT)	EIU	We do not find significant predictive power of democracy, media freedom and power distance on the speed of government responses.
Chiplunkar and Das (2021)	Global (125 countries)	How do countries with differing political institutions respond to national crises?	Aggregated index of containment and health policies (OxCGRT)	Polity IV (dummy)	Non-democracies impose more stringent policies prior to their first COVID-19 case, but democracies close the gap in containment policies and surpass non-democracies in health policies within a week. Democracies with greater media freedom respond more slowly in containment policies, but more aggressively in health policies.
Dempere (2021)	Global (156 countries)	National government success factors at controlling the first wave of COVID-19.	Stringency index, Outbreak response time, Testing rate	EIU, V-Dem (various indicators)	Countries with the highest democracy indexes applied the softest social constraints measured by the daily average stringency index. These countries exhibited the shortest outbreak response time and the most extensive daily average tests per thousand.
Engler et al. (2021)	Europe (34 countries)	Why some democracies were willing to constrain freedoms and concentrate power more than others during the first wave	OxCGRT, V-Dem (Pandemic Violations of Democratic Standards Index)	The Swiss National Center of Competence in Research (The Democracy Barometer)	In countries where the quality of democracy is higher in normal times, governments were more reluctant to adopt measures that are potentially in conflict with democratic principles.
Erić et al. (2021)	Europe (15 countries)	Impact of key economic and social variables in period of the first wave of COVID-19 pandemic on economic stimulus	COVID-19 Economic Stimulus Index	EIU	Democracy contributes to the economic policy response to pandemic in all three observed cases.
Lins et al. (2020)	Global (168 countries)	Do different political regimes react differently to COVID-19?	Time from the first confirmed case to adopting a strict social isolation measure (OxCGRT)	V-Dem (Regimes of the World Index) (dummy)	The political regime has no major influence. Democratic and autocratic regimes have similar performances in taking action to combat the disease.
Lundgren et al. (2020)	Global (180 countries)	Why have some states declared states of emergency when others have not?	Declaration of state of emergency (dummy)	V-Dem (Liberal Democracy Index)	Weak democracies with poor preparedness have been considerably more likely to opt for an SOE than dictatorships and robust democracies with higher preparedness.
Mietzner (2020)	Indonesia	Why was the outbreak first ignored and responded with piecemeal measures by the Indonesian central government?	Neglicence of risks, coherent policy action	Democratic controls over government, minority protection, treatment of opposition, corruption	Indonesia’s response was the result of its specific process of democratic decline in the last decade.
Rocco et al. (2021)	15 federal democracies	How subnational governments in federal democracies collect and report data on COVID-19 cases and mortality associated with COVID-19.	National scores on the Subnational COVID-19 Data Quality Index (four component indices)	V-Dem (Liberal Democracy Index,) V-Dem indicators (Subnational elections free and fair, Media independence)	The quality of subnational surveillance data in federations depends in part on public health system capacity, fiscal decentralization, and the quality of democracy.
Sebhatu et al. (2020)	OECD countries	The adoption of nonpharmaceutical interventions in the OECD countries during the early phase of the pandemic.	The day a general policy is adopted (OxCGRT)	V-Dem (Electoral Democracy Index)	Governments in countries with a stronger democratic structure are slower to react in the face of the pandemic but are more sensitive to the influence of other countries.

One key topic in this research concerns the type and timing of policy responses. Research on the type of policy responses deals largely with the issue of stringency and violations of democratic principles in connection with specific measures. Dempere [49] argues that countries with the highest democracy indexes (using various indicators from different indices) applied the softest social constraints measured by the daily average stringency index. These countries exhibited the shortest outbreak response time and the most extensive daily average tests per thousand. Chiplunkar and Das [50] show that non-democracies (Polity IV) impose more stringent policies (OxCGRT) prior to their first COVID-19 case, but democracies close the gap in containment policies and surpass non-democracies in health policies within a week of registering their first case. Democracies with greater media freedom respond more slowly in containment policies, but more aggressively in health policies. Engler et al. [51] find that in countries where the quality of democracy (Democracy Barometer) is higher in normal times, governments were also more reluctant to adopt policy measures (OxCGRT and PanDem) that are potentially in conflict with democratic principles. Lundgren et al. [52] study the declaration of a state of emergency. They find that weak democracies (V-Dem) with poor preparedness (GHS) have been considerably more likely to opt for a state of emergency than dictatorships and robust democracies with higher preparedness. Research on timing offers more mixed findings. Chen et al. [53] do not find significant predictive power of democracy (EIU) on the speed of government responses. However, Sebhatu et al. [54] shows that governments in countries with a stronger democratic structure (V-Dem) were slower to react in the face of the pandemic but were more sensitive to the influence of other countries.

The remaining articles in this category focus on more specific policy areas. The econometric analysis of Erić et al. [55] shows that democracy contributes to the economic policy response to pandemic, while Lins et al. [56] study the impact of the political regime type (V-Dem) to the adoption of stay-at-home requirements (OxCGRT) and find no major influence.

#### Epidemiological characteristics

Like in previous category, most research on the impact of democracy to the epidemiological characteristics of the pandemic are focused on the country level and address broad country groups (see Table 5). In fact, only Palguta et al. [57] discuss sub-national issues: they show that COVID-19 infections grew significantly faster in voting compared to non-voting constituencies in the Czech Republic.

**Table 5:** Articles discussing the impacts of democracy to the epidemiological characteristics of COVID-19.

Authors (year)	Geographic location	Topic area	Indicators / explanandum	Indicators / explanans	Conclusions
Achim et al. (2021)	Global (185 countries)	The influence of democracy upon the spread of COVID-19.	Case fatality rate, Infection rate, Mortality rate, Testing rate	EIU, V-Dem (Electoral Democracy Index, Liberal Democracy Index, Participatory Democracy Index)	We find that in high income countries, higher levels of democracy reduce the spread of COVID-19 while in the low income countries its influence is the opposite.
Annaka (2021)	Global (108 countries)	Relationship between political regimes, data transparency, and COVID-19 deaths	Mortality rate	Polity2, V-Dem (Multiplicative Polyarchy Index)	Authoritarian countries do not necessarily tend to have fewer COVID-19 deaths than their democratic counterparts. Data transparency is positively correlated with the number of death cases more consistently.
Chen et al. (2022)	Global (136 countries)	What factors might explain the cross-country variations in COVID-19 public performance?	Mortality rate, Infection rate	Policy stringency (OxCGRT), EIU as moderating variable	The negative effects of restrictive policies on infection and death rates are moderated by political trust and democracy levels. Under conditions of higher political trust and lower democracy levels, the policy effects on infection and death rates are greater.
Dempere (2021)	Global (156 countries)	National government success factors at controlling the first wave of COVID-19.	Mortality rate (daily, total), Infection rate	EIU, V-Dem (various indicators)	Countries with the highest democracy indexes suffered a more severe pandemic impact confirmed by the highest daily averages of cases and deaths per million and the highest mortality rate.
Huang et al. (2020)	Global (94 countries)	The association between previous exposure to SARS and/or MERS and the 30-day COVID-19 incidence rate.	Infection rate	EIU	Countries being classified as having “full democracy” using Democracy Index had higher incidence of COVID-19
Jain and Singh (2020)	Global (78–126 countries)	Socio-economic variables that determine a nation’s exposure to COVID-19 infections and deaths	CFR, Infection rate, Mortality rate, Testing rate	EIU	Democracy and good governance plays significant role in curtailing mortality rates. But there also takes place a rise in infected patients in the presence of democracy and higher per capita income.
Jardine et al. (2020)	Muslim majority countries (44 countries)	COVID-19 burden, epidemiology and mitigation strategies in Muslim-majority countries.	Infection rate, Mean estimated doubling time, Percentage of countries with flattened epidemic curves	EIU	Functional democracies were able to contain the epidemic significantly better than nondemocratic regimes.
Karabulut et al. (2021)	Global (99–128 countries)	Democracy measures and epidemiological characteristics of the COVID-19 pandemic	CFR, Infection rate	FH (Political Rights Index, Civil Rights Index, Global Freedom Score), V-Dem (Electoral Democracy Index), Polity	The infection rates of the disease appear as higher for more democratic countries, their observed CFRs are lower. There is a negative association between CFR and government attempts to censor media. However, such censorship relates positively to the infection rate.
Lago-Peñas et al. (2022)	Global (68–113 countries)	The role played by institutions at the country level in fighting the spread of Covid-19.	Mortality rate (accumulated)	FH (Political Rights Rating)	Our main results show that having either democracies or autocracies does not represent a crucial issue for successfully addressing the pandemic
Norrlöf (2020)	Global (139–157 countries)	This article traces the global spread of the virus scaled to population and CFRs of different countries.	CFR, Infection rate	FH (Global Freedom Score), V-Dem (Liberal Democracy Index)	Liberal democracies do not have higher cases per capita than other regime types according to any of the measures which could be used to characterize liberal democracy. However, liberal democracies have a higher CFR than other regime types.
Palguta et al. (2022)	Czech Republic	We examine whether large-scale, in-person elections propagate the spread of COVID-19.	Infection rate (cumulative & active cases), Hospitalization, PCR test positivity rates	Voter turnout	New COVID-19 infections grew significantly faster in voting compared to non-voting constituencies in the second and third weeks after the elections.
Serikbayeva et al. (2021)	Global (137–141 countries)	The effects of state capacity on the Covid-19 CFRs.	CFR	FH (Global Freedom Score)	The effect of democracy level on the Covid-19 death level is statistically significant for non-free countries in the models controlling for government effectiveness and the testing and stay at home policies. In non-free countries the likelihood of a higher death rate is lower compared to free countries.
Sorci et al. (2020)	Global (67–143 countries)	Identification of key factors possibly explaining the variability in CFR across countries	CFR	Polity IV	Moderate evidence suggesting that countries with a democratic regime were those with the highest CFR.
Vadlamannati et al. (2021)	Global (210 countries)	Whether an ‘egalitarian democracy’ generates favourable outcomes regarding the COVID-19 pandemic	Mortality rate, Testing rate	V-Dem (Egalitarian Democracy Index)	More equitable access to health care increases testing rates and lowers the death rate from COVID-19. Broader egalitarian governance, measured as egalitarian democracy shows the opposite effect.
Yao et al. (2022)	Global (148 countries)	The influence of democracy and other factors on the CFR of COVID-19 during the early stage of the pandemic	CFR, Testing rate, Cumulative cases, Cumulative deaths	EIU	The findings suggest that a higher Democracy Index is associated with more deaths from COVID-19 at the early stage of the pandemic (in 47 high-income countries), possibly due to the decreased ability of the government.

The key issues addressed here are the impacts of democracy to COVID-19 cases, deaths, and case fatality rates (CFR; i.e., proportion of people diagnosed with a certain disease and end up dying of it over time). Most studies associate democracy with higher levels of COVID-19 incidence globally. Using various indices, Dempere [49] and Karabulut et al. [58] show that countries with the highest democracy index scores suffered a more severe pandemic impact. Higher levels of incidence are especially found among countries being classified as having “full democracy” by the EIU Democracy Index [59, 60]. Similar findings can be found from narrower country groupings. For example, Jardine et al. [61] find that non-democratic regimes had much shorter doubling time of cases compared to functional democratic Muslim-majority countries.

Others suggest that the relation between the extent of democracy and COVID-19 incidence is not linear but is shaped by numerous moderating factors. For example, Achim et al. [62] find that in high-income countries higher levels of democracy (as measured by EIU and various V-Dem indices) reduce the spread of COVID-19 while in the low-income countries its influence is exactly the opposite. Chen et al. [63] show that democracy levels (EIU) moderate the effects of policies on infection and death rates (OxCGRT).

Research on the relation between democracy and COVID-19 deaths offers varied findings. Lago-Peñas et al. [64] find that the coefficient between the extent of political rights and COVID-19 deaths is negative and statistically significant but only for estimates using accumulated data up to September 2020. Annaka [65] shows that authoritarian countries do not necessarily tend to have fewer COVID-19 deaths than their democratic counterparts (as defined by Polity and V-Dem indices). Vadlamannati et al. [66] suggest that more equitable access to health care increases testing rates and lowers the mortality rate from COVID-19, but egalitarian democracy (V-Dem) shows the opposite effect.

Research on CFR has provided different findings over time. Research on the early stages of the pandemic associate democracy with higher CFR. Using the Polity IV index, Sorci et al. [67] found moderate evidence suggesting that countries with a democratic regime were those with the highest CFR. Norrlöf [68], using the FH index, finds that liberal democracies have a higher CFR than other regime types (although liberal democracies do not have higher cases per capita than other regime types). Serikbayeva et al. [69] find that the level of democracy (FH) has a statistically significant positive impact on CFR in non-free countries, and that the likelihood of a higher death rate is lower in non-free countries compared to free countries. Yao et al. [70], using the EIU index, suggest that a higher Democracy Index is associated with (and moderated by increased hospital beds and healthcare workforce per capita) more deaths from COVID-19 at the early stage of the pandemic in all countries. However, later research offers somewhat different results. Karabulut et al. [58], using various indices (FH, Polity, and V-Dem), show that the observed CFR are in fact lower for democratic countries in a longer time period.

### The impacts of the pandemic to democracy

Research on the pandemic’s impacts on democracy requires some further tools for interpretation. We use Dryzek’s [26] three dimensions of democracy – scope, franchise, and authenticity – to map out different types of impacts to democracy. The three dimensions are not dependent on a particular theory or model of democracy but can be applied across different conceptions of democracy. *Scope* refers to the extent to which different areas of life are under democratic control. *Franchise* refers to the effective number of participants who exercise influence over a democratic decision. *Authenticity* denotes the degree to which democratic control is substantive (rather than symbolic) and engaged by competent (rather than incompetent) and reflective (rather than inconsiderate) actors.

Most research insights presented in the sample deal with only one of the three dimensions. Some (albeit few) papers discuss more than one dimension, and thus appear more than once in the following sections. The two exceptions that escaped our attempts to categorize their insights are the paper that introduces the PanDem index [31] and another paper that discusses the state of democracy in the world in 2020 [30]. These papers do not attribute the violations of democracy or the state of democracy to any one specific conception of democracy but to more general principles that are relevant to various conceptions of (liberal) democracy. The problem here is that the violation or enactment of principles can be interpreted differently depending on how exactly democratic politics is understood. For example, where one theory of democracy that focuses on citizen rights might regard the curtailing of freedom of movement as a curtailment of the scope of democracy, another theory that focuses on participation might regard it as a curtailment of democratic franchise. To avoid such conflations, we have excluded the papers from our analysis in this section. Their more general conceptual and theoretical contributions have been presented above.

#### Scope

Most papers belonging to this category focus on democratic institutions of decision-making (see Table 6). There are only two exceptions here, one focused on freedoms and another on political rights. Cassani [71], who studies the impacts of the policy responses to COVID-19 to citizen freedoms, finds a widening freedom divide between autocratic and democratic regimes. Kinowska-Mazaraki [72] shows that Poland curtailed the right of assembly and protest, hence limiting the scope of democratic action.

**Table 6:** Articles discussing the impacts of the COVID-19 pandemic to the scope of democracy

Authors (year)	Geographic location	Topic area	Focus area/indicators	Conclusions
Andersson and Aylott (2020)	Sweden	Comparison of the coronavirus strategy to previous episodes of Swedish policy exceptionalism.	Expansion of policy-making without democratic accountability	Policy was being shaped not by the government, but by public agencies led by strong-willed chief executives.
Cassani (2021)	Global	A review of the literature on the short-term impact of the pandemic on citizen freedoms	Citizen freedoms; 4 freedom indices (V-Dem)	Democratic and autocratic regimes have dealt with the pandemic in quite different ways, leading to the widening of the freedom divide between these forms of political regime.
Gamkrelidze (2022)	Georgia	Why the state emergency was damaging for democracy in Georgia	Legal vs. non-legal order, visibility of political opponents	The state of exception further flattened political diversity through limiting the space for political pluralism.
Guasti (2020)	CEE (Visegrad)	The COVID-19 pandemic as a stress-test for the already disrupted liberal-representative democracies.	Degree of technocracy, triggers to democratic resilience	The pandemic accentuates the existing democratic disfigurations. In Hungary and Poland, the populist leaders instrumentalized the state of emergency to increase executive aggrandizement. In the Czech Republic and Slovakia, democracy proved resilient.
Guasti (2021)	Visegrad	Under what conditions does executive dominance turn into executive aggrandizement?	Vertical, horizontal, and diagonal political accountability	Democracy eroded in Hungary and, to a lesser degree, in Poland but remained resilient in the Czech Republic and, to a lesser degree, in Slovakia.
Hallock and Call (2021)	El Salvador	Whether pandemic response policies undermine or affirm democratic controls on state leaders and institutions	Expansion of policy-making without democratic accountability	El Salvador represents a unique case where citizens appear to have rewarded their head of state for defying democratic checks on his power to implement proactive and restrictive health measures.
Kinowska-Mazaraki (2021)	Poland	The shift from democratization to the opposite direction in Poland	Public protests	The COVID-19 pandemic has given the ruling party a reason to further limit the right of assembly and protest.
Lewkowicz et al. (2022)	Global	The drivers of democracy backsliding during the COVID-19 pandemic	PanDEM and V-Dem indices; rule of law; demographic and economic covariates; COVID-19 incidence rate	The stronger the rule of law and the higher levels of (electoral) democracy, the lower the risk of democracy backsliding in the face of the global pandemic.
Lozano et al. (2021)	Canada, Australia, New Zealand and the UK	The performance of democratic accountability mechanisms in four parliamentary democracies.	Index with 9 components of democratic accountability	When democracies have already established robust accountability mechanisms before a crisis, they are more likely to maintain high accountability standards and resist actions that deviate from regular practice.
Mills (2019)	Australia	Emergency response to the pandemic in Australia.	Balance of powers and the scope of parliamentary accountability	Parliament’s authorising and deliberative functions were expedited and, with adjournment, then terminated. Parliament’s accountability function was saved from elimination by the Senate’s capacity to install a mechanism for all-party scrutiny of executive decision making.
Passos and Acácio (2021)	Latin America	Civil-military balance	Delegation of non-military missions to the military	Latin American democracies have without exception militarized to some degree their response to the pandemic.
Prakash (2021)	India	Étatisation and suspension of politics, in India.	Expansion of executive power	Management of the pandemic in India has reinforced longer-term trends of étatisation and insulation of the executive from accountability.
Sebastião (2021)	EU/EMU	Has the democratic deficit of the decision-making in the Eurozone crisis response repeated in the COVID-19 response?	Constitutionally mandated scope of democratic decision-making	Despite the different results, the institutional status quo hasn’t changed.
Setijadi (2021)	Indonesia	Indonesian COVID-19 response and its impacts to the democratic regression of the country.	Scope of executive powers	Laws that restrict freedom of speech and the further empowerment of the military and intelligence agencies in civilian life have allowed for further democratic regression.

The remaining twelve papers of this category deal with the state of exception or related aspects of the expansion of executive powers and limiting of democratic accountability and deliberation. A few papers discuss ‘executive aggrandizement’ [73] during the pandemic. In some countries like Australia [74], the democratic accountability of the executive was (due to popular protests only temporarily) abandoned to provide leeway for the making of pandemic response policies. Some observe that technocratic policymaking by public health officials [73, 75] replaced democratic procedures in pandemic responses. Others observe a similar tendency in the case of the military [76].

A significant number of papers in this category argue that the pandemic aggravated the already ongoing and more general expansion of executive powers to replace previously democratic politics in ‘democratically backsliding’ countries. The argument has been made in the cases of El Salvador [77], Georgia [78], Hungary [79], India [80] and Indonesia [81]. Others have found that the pandemic has not deepened existing democratic deficiencies. This case has been made the European Union [82] and some individual countries like the Czech Republic and Slovakia [73, 79]. Lewkowicz et al. [83], utilizing the V-Dem indices and the PanDem index, show that the stronger the rule of law and the higher levels of electoral democracy, the lower the risk of democratic backsliding has been in the face of the pandemic. Previous strengthening of democratic accountability mechanisms has also been found to decrease the likelihood of democratic backsliding [84].

#### Franchise

All papers in this category discuss the impacts of the pandemic to elections (see Table 7). The difficulty of holding elections under a pandemic have been widely noted. Only a case study on Israel shows that election turnout can be maintained through effective containment procedures, logistics, and communications [85]. Otherwise, the articles of this category observe a decreasing voter turnout during the pandemic. The countries in which this has been observed include Chile [86], Ghana [87], Ethiopia and Mali [88], and India, Pakistan and Afghanistan [89]. Some also argue that the pandemic has halted the efforts to instill democratic elections [90].

**Table 7:** Articles discussing the impacts of the COVID-19 pandemic to the franchise of democracy

Authors (year)	Geographic location	Topic area	Focus area/indicators	Findings
Afek et al. (2020)	Israel	General elections for the 23rd Knesset during the COVID-19 pandemic	Electoral turnout	The high rate of participation in elections was the result of early effective containment, effective communications to reassure the general public on voting safety, legislation, and logistics measures.
Ayandele et al. (2021)	Burundi	How the pandemic and government-adopted measures to curb the spread of the virus have given room for abuse of democratic processes	Interest and monitoring capacity of election processes	COVID-19 pandemic affects pre-and post-election processes by undermining efforts to instill sustainable democratic practices such as elections.
Kumi (2022)	Ghana	Impact of the COVID-19 pandemic to civic space and elections	The scope of political rights and voter turnout	The implementation of legislations resulted in unintended consequences characterized by restrictions of civil liberties including freedoms of movement, expression, association, and peaceful assembly which threatened the civic space.
Matlosa (2021) [88]	Africa (Mali and Ethiopia in more detail)	The impact of the government responses to pandemic on elections	Free multi-party elections	Some countries have proceeded with elections, while others postponed their elections. Some elections have been marked by low voter turnout. International observers have been conspicuous by their absence in some elections.
Morales Quiroga (2021)	Chile	To what degree did the COVID-19 pandemic influence the development of the constitutional referendum of October 2020?	Electoral turnout	The lower-income sectors, whose infection rates were higher than the rest of the population, turned out to vote in greater numbers than in previous elections
Nelson (2021)	India, Pakistan, Afghanistan	Social exclusion of Muslims and its impacts to electoral legitimacy	Electoral legitimacy; social inclusion/exclusion	COVID-19 has exacerbated and perhaps accelerated key trends. Exclusionary rhetoric and appeals to emergency powers have challenged the principles of democracy.

#### Authenticity

Three common themes can be found among the papers included in this category (see Table 8). First and the most common theme addressed here is related to democratic legitimation and justification of policymaking under the pandemic. Research suggests that some countries legitimized the expansion of executive powers democratically (e.g., Portugal [35]), whereas some others did not (e.g., India [91]). Mixed interpretations have been made regarding the Italian case [35, 92]. In some countries like Israel, the lack of democratic justification for executive aggrandizement led to wide popular backlashes, hence demonstrating democratic resilience [93]. But in Germany, a similar backlash did not occur, which raises questions about the degree of authenticity and resilience in the country [94]. Some have also addressed the preconditions for democratic emergency politics. Truchlewski et al. [95] argue that, by ‘buying time’ through effective emergency politics the EU enabled its member states time to democratically deliberate upon and justify their policy responses.

**Table 8:** Articles discussing the impacts of the COVID-19 pandemic to the authenticity of democracy

Authors (year)	Geographic location	Topic area	Focus area/indicators	Findings
Avritzer and Rennó (2021)	Brazil	How regime legitimacy, authoritarian attitudes, and support for a populist, authoritarian leader interact and are affected by the pandemic	Legitimacy of democractic rule	The pandemic did not contribute to the deepening of a democratic crisis among the Brazilian public
Baekkeskov et al. (2021)	Denmark, Sweden	Have national discourses represented arguments for policy alternatives evenly or skewed in favour of national policy?	Pluralist and balanced deliberation on policy options	Discourses on early COVID-19 responses tended toward monotony rather than pluralism. Whether leadership was epistemic or political, it took the form of repeating reasons for selected policies, rather than encouraging public debate over options.
Bar-Siman-Tov (2020)	Israel	How the pandemic challenges parliaments in countries where COVID-19 coincides with a pre-existing political crisis.	Democratic justification for the use of emergency powers	The Israeli democracy survived the dual challenge of political and COVID-19 crises.
Bohler-Muller et al. (2021)	South Africa	Do ordinary South Africans support the limitation of their rights?	Legitimacy of democracy	The Covid-19 Democracy Survey served as one way to facilitate democratic participation as it allowed people to express their views, opinions and concerns about the virus and living under lockdown.
Bol et al. (2021)	15 Western European countries	The political effect of the enforcement of a strict confinement policy in response to the pandemic.	Public support for democracy	Lockdowns have increased support for the status quo decision makers, institutions and regimes.
Casero-Ripollés (2020)	United States	How the pandemic has conditioned the dynamics of the media system and how it has affected democracy.	Media consumption as proxy for equality and accessibility concerning public affairs	The resurgence of the role of legacy media have in part reduced existing inequalities regarding news consumption.
Corradetti and Pollicino (2021)	Italy	Is the COVID-19 pandemic changing the constitutional power structures of democracies?	Constitutional justification for a state of exception	The emergency use of the power arises within an already established framework of constitutional justification.
De Angelis and de Oliveira (2021)	Italy, Portugal	Assessment of the institutional resilience of consolidated democracies in emergency situations	Checks and balances over a declaration of state of exception	Italian institutional and constitutional order falls short of a number of the criteria, because the regulation ends up overhauling normal checks and balances; the Portuguese constitutional order seems to pass the test of our criteria
Einstein et al. (2022)	Boston (US)	The representativeness of broader communities of public online meetings	Representativeness	Participants in online forums are quite similar to those in in-person ones. They are similarly unrepresentative of residents in their broader communities.
Elstub et al. (2021)	UK	Analysis of the deliberative capacity of citizens in a pandemic.	Citizen participation in and quality of online deliberation	Our evidence indicates that deliberation can be resilient in a crisis.
Ferry et al. (2021) [119]	United Kingdom	How the UK government used data to legitimate policy and support implementation	Parliamentary and public ability to understand government decisions and hold them to account	The data connected the government to the governed and enabled democratic accountability.
Ghosh (2021)	India	The process of policy-communication on the pandemic	Degree of democratic accountability	Government’s several omissions and commissions have defied the norms of democratic accountability
Kwan (2021)	Singapore	Youth motivations, participation forms and how participation shapes future sociopolitical engagement.	Participation in election work	Singaporean youth were motivated to build awareness and activism and take action between elections and during GE2020.
Lupu and Zechmeister (2021)	Haiti	Whether and how the appearance of the pandemic would shift public opinion toward the president, elections, and democracy.	Popular legitimacy of democratic rule	The pandemic moved in a manner consistent with the kinds of rally effects documented in contexts of interstate conflict. We find no evidence of a broader shift in democratic attitudes.
Matlosa (2021) [120]	Africa	The crisis of international election observation in Africa during the pandemic	International election observation	The onset of COVID-19 has compounded the crisis of international election observation.
Merkel (2020)	Germany	Reflections on the implications of state of exception as mode of governance on institutions and actors of democracy.	Declaration of state of exception	We cannot rule out longer-term habituation effects of temporary authoritarian rule among the citizens in the near future.
Pedrazzani et al. (2021)	Italy	The degree to which citizens perceive democratic institutions as effective in coping the emergency.	Legitimacy of democratic rule, perceptions of democratic performance	Evaluations of democracy became more negative with social proximity to the disease and with individual perceived vulnerability, understood in health and economic terms
Truchlewski et al. (2021)	EU	Recognizing an emergency politics that buys time for democracies	Ability to sustain democratic decision making during exceptional times	The European Commission bought time for member state governments to deliberate.

Another theme concerns the democratic virtues and vices of new communication technologies that popularized during the pandemic. Some positive impacts to authenticity are observed here. New online forms of election work are observed to have activated the youth to participate in election work in Singapore [96] whereas online scientific surveys offered South Africans a way to express popular views under lockdowns [97]. Anecdotal evidence suggests that even though online platforms may maintain or even enhance the quality of deliberation [98], they may also be unrepresentative of the broader communities [99]. Another discussion concerns the role of traditional information sources for democratic actorhood. Casero-Ripollés [100] observes that legacy media consumption surged in the United States during the pandemic. However, Baekkeskov et al. [101] also note that media discourses became much less deliberative and more monotonous during the pandemic.

The third common theme concerns the support for democratic politics. Here, the evidence is highly varied. Bol et al. [102] find that lockdowns increased satisfaction with democracy in Western Europe. But in the case of Italy, Pedrazzani et al. [103] report that evaluations of democracy became more negative with social proximity to the disease and with individual perceived vulnerability. Despite observing the rally effects documented in contexts of interstate conflict, no evidence of a broader shift in democratic attitudes due to the pandemic can be observed in Brazil [104] or Haiti [105].

## Discussion

The early research on democracy and the COVID-19 pandemic offers a diverse body of literature. Our topic modeling suggests that the scholarship that mentions the two concepts deals with various issues: the nature of crises brought by the pandemic, epidemiological characteristics, political behavior, the governance of responses to the pandemic, the (temporary or longer-term) narrowing down of citizen rights amidst the pandemic, the virtues and vices of new technologies, and societal challenges and change. 94 articles discussed the relation between democracy and the pandemic more systematically. The body of papers with conceptual and theoretical contributions in this sample has offered new insights on the possibilities, difficulties, and means to maintain democracy under severe health crises such as pandemics. This research has given rise to new indices to track violations of democratic principles in crises, new criteria for governing rapid policy responses democratically, new ideas on how to organize elections under health crises, and warnings about the longer-term impacts of new policies, technologies, and discourses with anti-democratic qualities.

Empirical research on democracy’s impact on the COVID-19 pandemic and vice versa also offers a versatile body of research. We find that the methodologies used, the geographical scope of research, and the scholarly contributions vary according to the direction of influence studied. Research on democracy’s impacts on the epidemiological characteristics of and policy responses to the pandemic are largely based on democracy indices, country-group-level analysis, and varying timeframes. Democracy appears to have a significant impact on some aspects policy responses and epidemiological characteristics of pandemics. Be it about timing of policy measures, preferred types of measures, or preferences over the stringency of measures, democratic countries are likely to produce responses that somewhat differ from non-democratic countries as well as from each other. Democratic and non-democratic countries do not necessarily perform in a vastly different degree in dealing with pandemics in the short run. Beyond these observations, the results are somewhat mixed depending on the democracy indices, epidemiological and policy indicators, and time periods studied. Hence, further empirical research and meta-analyses are needed to say anything conclusive about democracy’s impacts on the pandemic. In-depth case studies and qualitative research is needed for theory-building.

Research on the pandemic’s impacts on democracy are largely based on qualitative research and discuss relatively few countries at a time. Many gaps still exist. For example, the impacts of the pandemic to democratic participation in the civil society and the quality of deliberation and representativeness in policymaking contexts were not explored systematically in the body of literature scoped here. Longer-term time series are needed to study the pandemic’s impacts on democracy globally.

Thus far, most findings concerning the impacts of COVID-19 to democracy raise some concern. In most parts of the world, the scope of democracy narrowed down due to the pandemic, albeit in most cases only temporarily. But in the already democratically backsliding countries, the pandemic offered new conditions for broadening executive powers time- and scope-wise beyond what may have been necessary to tackle the pandemic. The evidence concerning the franchise of democracy is very much limited to elections and election turnout, but it suggests that policy responses to the pandemic will have a major impact on the conduct of elections. Much is needed to maintain high degrees of democratic franchise. The authenticity of democratic politics has been compromised in various ways in the COVID-19 pandemic. Most importantly, the pandemic revealed alternatives to democratic politics. While the popular support for democratic politics decreased during the pandemic in some democratic countries, others broadly mobilized against policies without democratic justification. While new communication technologies are no panacea for maintaining authenticity, online channels may offer some opportunities to renew it.

## Conclusions

If the conceptual and theory papers reviewed above have offered important insights and hypotheses for further research, then the empirical research reviewed gives equally important reasons to keep a close eye on future events and test the hypotheses. Many papers have argued that the pandemic has accentuated different forms of democratic backsliding but is unlikely to have undermined democracy as such thanks to various mechanisms from constitutional checks and balances to popular backlashes that have proven the resilience of established democracies. Yet, empirical research shows some weak signals of antidemocratic tendencies that may become more accentuated in the longer run, ranging from the emergence of anti-democratic discourses to positive popular reactions to authoritarian forms of governance. Thus, it remains to be seen whether the longer-term impacts of the COVID-19 pandemic will eventually prove detrimental to democracy, and whether democracy will remain as resilient in the next large-scale health crisis as it did under COVID-19.

## Data Availability

All data generated and analyzed for the review are available upon request from the authors. The data can be made available upon reasonable request from the Corresponding author.
